# Interstitial Pregnancy Treated with Mifepristone and Methotrexate with High Serum β-hCG Level in a Patient Wishing to Preserve Fertility: Time to Define Standardized Criteria for Medical/Surgical Therapy?

**DOI:** 10.3390/ijerph191811464

**Published:** 2022-09-12

**Authors:** Felice Sorrentino, Lorenzo Vasciaveo, Vincenzo De Feo, Erika Zanzarelli, Elvira Grandone, Guglielmo Stabile, Luigi Nappi

**Affiliations:** 1 Department of Medical and Surgical Sciences, Institute of Obstetrics and Gynecology, University of Foggia, 71121 Foggia, Italy; 2 Thrombosis and Haemostasis Unit, Fondazione IRCCS “Casa Sollievo della Sofferenza”, 71013 San Giovanni Rotondo, Italy; 3 Ob/Gyn Department of the First I.M. Sechenov Moscow State Medical University, 119991 Moscow, Russia; 4 Department of Medicine, Surgery and Health Sciences, University of Trieste, 34100 Trieste, Italy

**Keywords:** non-tubal ectopic pregnancy (EPs), interstitial pregnancy, medical treatment, mifepristone, methotrexate, β-hCG, cornuostomy

## Abstract

Interstitial pregnancy (IP) accounts for 2% of all ectopic pregnancies and has a mortality rate of 2–2.5%. The diagnosis is made by a transvaginal ultrasound and the treatment can be medical or surgical. We report the case of a 36-year-old primigravida who was 6 + 5 weeks pregnant, diagnosed with interstitial pregnancy by ultrasound, who had a very high serum β-hCG level (31,298 mIU/mL) and wanted to preserve her fertility. The patient was treated with one dose of mifepristone and a double dose of methotrexate since the decrease in the β-hCG serum level was less than 15% after the first dose. At the beginning, medical therapy was effective, as no embryonal cardiac activity was detected and serum β-hCG levels decreased early, but on the 20th day of hospitalization, the patient underwent surgery for her clinical symptoms and the evidence of free fluid in the Douglas pouch at a transvaginal ultrasound exam. Our experience showed that medical treatment should be considered, especially in women wishing to preserve their fertility. Further studies are needed to establish a standardized protocol and maybe a clinical score that can be useful in predicting the patients in which medical therapy could be most successful.

## 1. Introduction

Non-tubal ectopic pregnancy (EPs) account for fewer than 10% of all EPs [1]. Interstitial pregnancy (IP) is a rare form of ectopic pregnancy (EP) characterized by the implantation of a blastocyst in the interstitial part of the fallopian tube, which invades the myometrium [2]. The incidence of IP accounts for 2% of all ectopic pregnancies and it is the form of EPs with the highest risk of mortality due to the possibility of uterine rupture, leading to massive hemorrhage, hemoperitoneum, hypotension and shock. It is important to distinguish an IP pregnancy from an eccentrically implanted intrauterine pregnancy (IUPs) so that it can be managed differently. The mortality rate is 2–2.5% because of misdiagnosis of these gestations as IUPs. Several risk factors may play a role, such as previous ectopic pregnancy or pelvic inflammatory disease (PID), uterine abnormalities, previous salpingectomy or pelvic surgery, or the use of assisted reproductive techniques (ART) [3]. Contrary to previous belief, rupture of interstitial pregnancy occurs relatively early in pregnancy and symptoms include vaginal bleeding and abdominal pain [4]. Diagnosis is made by transvaginal ultrasound (TVUS), which is the gold standard [4]. Because of the rarity of the disease, there is limited evidence on which is the best option for a safer management, and the evidence is mostly based on small case series reported in the literature. It depends on several factors, the greatest of which is the serum level of beta-human chorionic gonadotropin (β-hCG), even if cases of successful medical treatment with high β-hCG serum levels have been reported in the literature [5]. Other important factors are the size of the amniotic sac and the patient’s desire to preserve fertility. Medical therapy should only be considered if the patient is hemodynamically stable. It consists of methotrexate (MTX) with local injection or oral administration and single or multiple doses. Mifepristone can be given in a single oral dose. Surgery is necessary in patients who are not hemodynamically stable and in case of failed medical treatment. The most effective surgical treatments are laparoscopic unilateral cornuotomy and laparoscopic unilateral salpingectomy [1,6]. We present the case of a woman with an interstitial pregnancy with high serum β-hCG levels (31,298 mIU/mL) who was treated with methotrexate and mifepristone to preserve her fertility.

## 2. Case Presentation

We describe the case of a 39-year-old primigravida who sought medical help in the Gynaecology and Obstetrics Department of the University Hospital of Foggia.

The patient was 6 weeks and 5 days pregnant and complained of abdominal pain and vaginal spotting that had started two days earlier. This pregnancy was induced by assisted reproductive technologies. The patient had a history of a previous ectopic pregnancy with right salpingectomy. Physical examination and vital signs were normal except for the reported abdominal pain.

**Figure 1:**
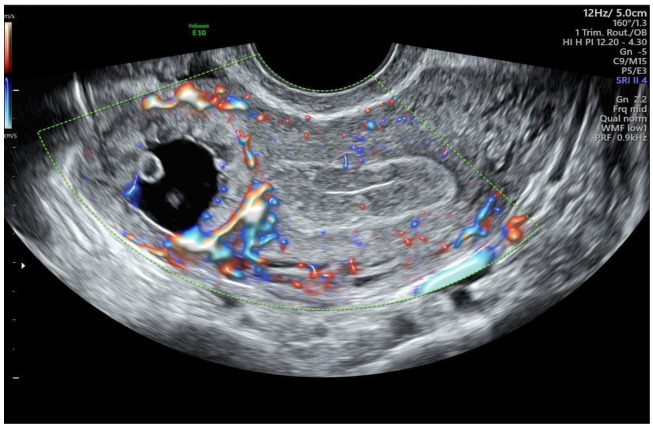
Transvaginal ultrasound finding of interstitial pregnancy.

**Figure 2:**
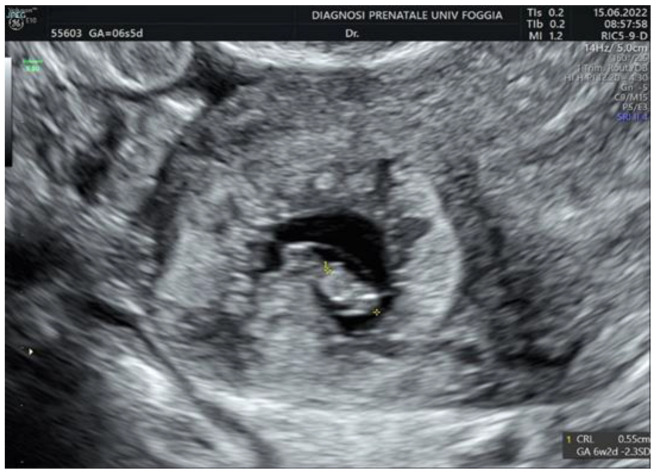
Embryo located eccentrically in the right side of the uterine fundus.

**Figure 3:**
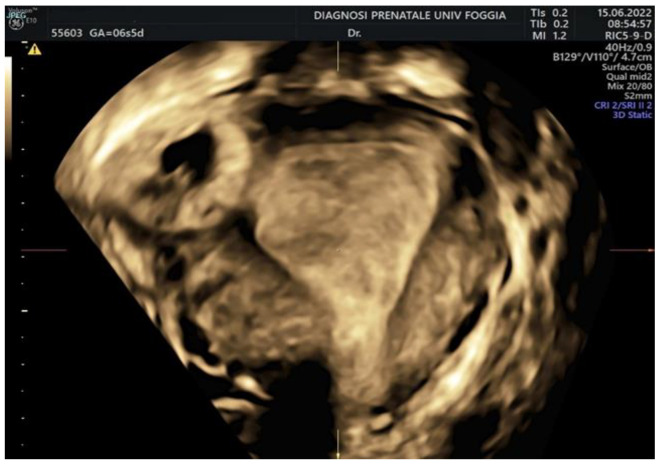
3D transvaginal ultrasound showing the interstitial pregnancy.

Then, a transvaginal ultrasound scan was performed by an experienced sonographer with a transvaginal probe (Voluson E10 BT19 ultrasound machines, GE healthcare, WI, Chicago, IL, United States). The exam revealed a 15 mm gestational sac with a 5.5 mm embryo with cardiac activity located eccentrically in the right side of the uterine fundus, in the right interstitium, surrounded by a subtle myometrium, suggestive of an interstitial pregnancy (Figure 1, Figure 2 and Figure 3).

**Figure 4:**
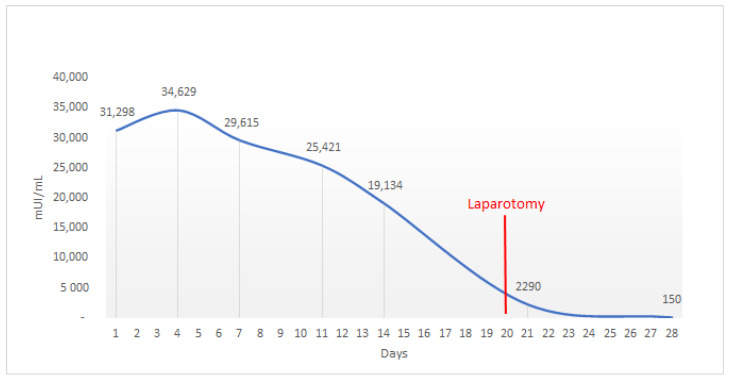
ꞵ-hCG serum level during hospitalization. On day 1 first methotrexate dose was administered, the second one was given on day 7.

**Figure 5:**
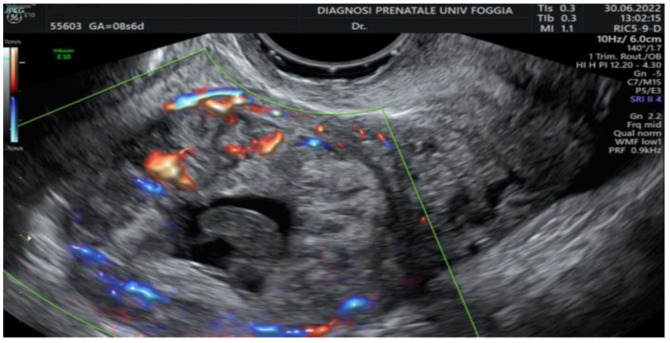
Transvaginal ultrasound control performed on day 15.

**Figure 6:**
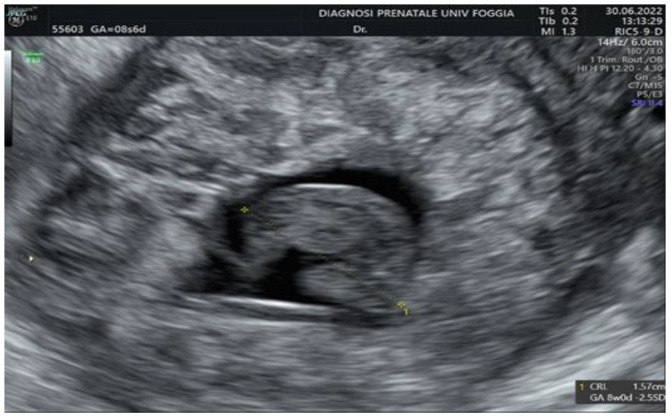
Embryo with no cardiac activity on day 15.

**Figure 7:**
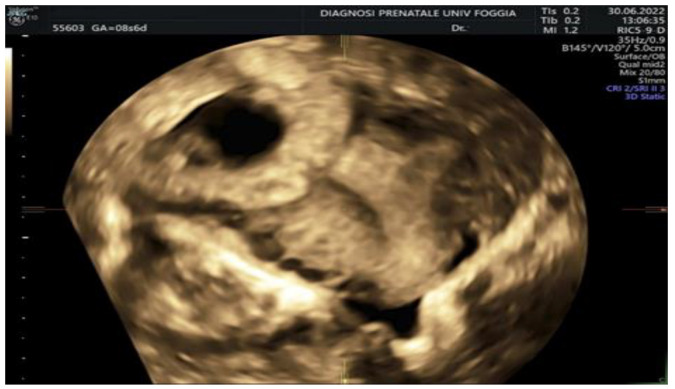
3D transvaginal ultrasound scan performed on day 15 showing the interstitial pregnancy.

Haemoperitoneum was excluded. The serum level of human chorionic gonadotropin at the time of admission was 31,298 mIU/mL. The patient was diagnosed with right-sided interstitial pregnancy. The patient was given appropriate and extensive counseling and very detailed information about therapeutic strategies, comparing the risks and benefits of medical and surgical treatments. In addition, the patient was widely informed that failure of medical therapy or a change in clinical condition (hemodynamic instability) could result in a transition to surgical therapy with possible emergency laparotomy access. The patient signed an informed consent to allow anonymized data collection for research purposes. All procedures performed in the study were in accordance with the ethical standards of the institutional research committee and with the 1964 Helsinki declaration and its later amendments or comparable ethical standards. A dose of methotrexate was calculated based on the estimated body surface area using the DuBois method. A single intramuscular dose of 90 mg methotrexate (50 mg/m^2^) was administered along with a single dose of 600 mg mifepristone taken orally. The serum level of human chorionic gonadotropin initially increased from 31,298 mIU/mL on day 1 to 34,629 mIU/mL on day 4 and then decreased to 29,615 mIU/mL on day 7. Ultrasound examination showed an equal-sized amniotic sac containing a 10 mm embryo with cardiac activity. As the decrease in serum β-hCG level was less than 15%, we decided to administer a second dose of 90 mg methotrexate (Figure 4). The serum level of β-hCG decreased rapidly to 25,421 mIU/mL on day 11 and 19,134 mIU/mL on day 14, and ultrasound scans performed on day 15 revealed an increasingly small gestational sac with the absence of embryonal cardiac activity (Figure 5, Figure 6 and Figure 7).

**Figure 8:**
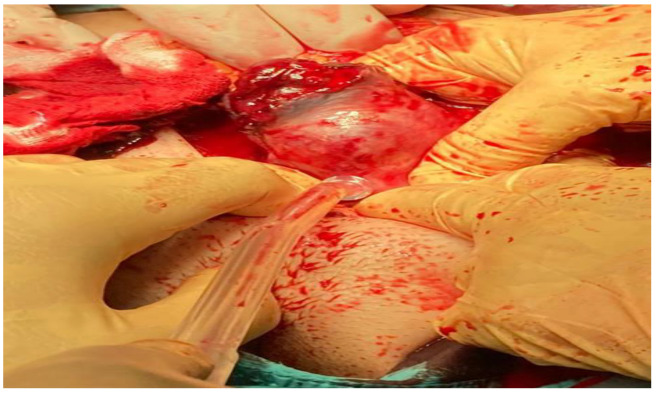
Laparotomic view of cornuectomy after interstitial pregnancy uterine rupture.

The patient remained asymptomatic for 19 days. On the 20th day of hospitalisation, the patient suddenly complained of severe stabbing abdominal pain. Immediately, a transvaginal ultrasound examination was performed, which revealed free fluid in the Douglas pouch. Due to clinical haemodynamic instability, we decided on a surgical strategy and the patient underwent an urgent laparotomy: right cornuectomy with removal of the interstitial pregnancy and surgical toilet were performed (Figure 8). The operation was performed by an experienced gynaecological surgeon with special skills in laparotomy. In total, 1000 cc of haemoperitoneum was drained. The patient was discharged 3 days later. Postoperative follow-up was scheduled for 30 days after surgery. Physical examination, transvaginal ultrasound and serum levels of β-hCG were all normal.

## 3. Discussion

Interstitial pregnancy is a rare condition that accounts for about 2–6% of all ectopic pregnancies and is characterized by an embryo implanting in the interstitial part of the fallopian tube [7,8]. However, the more common use of assisted reproductive technologies (ART), including ovulation induction, intrauterine insemination (IUI), in vitro fertilization (IVF) and intracytoplasmic sperm injection (ICSI), is leading to an increase in extrauterine pregnancy [9]. Other risk factors include previous tubal pregnancies, tubal surgeries, a history of pelvic inflammatory disease, and sexually transmitted diseases [1]. These risk factors are similar to those for other tubal pregnancies, except for ipsilateral salpingectomy, which is a risk factor specific for interstitial pregnancy. Diagnosis is based on clinical presentation, serum β-hCG level and sonographic criteria: an empty uterine cavity, gestational sac located in the interstitial part of the tube and surrounded by a thin myometrium, the interstitial rail sign (an echoic line connecting the gestational sac to the uterine cavity) and increased vascularisation on color Doppler ultrasound [10]. An MRI scan may be considered if ultrasound is insufficient for diagnosis [11]. Thanks to the use of increasingly precise, high-resolution instruments, diagnosis can be made promptly and more easily [12]. Interstitial pregnancies are characterized by high risk of severe hemorrhage and maternal morbidities. Early diagnosis of an ectopic pregnancy makes it possible to reduce the number of obstetric emergencies, which can be life-threatening for the pregnant woman. It has been reported that surgical treatment of an interstitial pregnancy diagnosed in time has a success rate of 85–90% [5]. A clear consensus on the management of interstitial pregnancy has still not been reached and no clear guidelines have been established. Nevertheless, the rise in quantitative serum β-hCG levels and advances in TVUS allow physicians to follow up and diagnose interstitial pregnancy at an earlier gestational age [13]. In the literature, no precise data about serum β-hCG level trends are gathered; thus, it cannot be the only key element that leads to a diagnosis and medical or surgical approach [2]. Evidence shows that medical treatment for ectopic pregnancies may not be sufficiently efficacious; hence, surgical approach, although more invasive, may be preferable for its success rate. Previous ectopic pregnancy has been highlighted as a critical risk factor for failure of medical therapy in interstitial pregnancies [14]. In cases of haemodynamic stability, medical treatment may be considered to preserve fertility. The size of the gestational sac and the thickness of the surrounding myometrium are other factors that need to be considered [13]. There are several options for treatment. Medical therapy is based on the intramuscular administration of methotrexate. According to the Stovall protocol, the serum-β-hCG level must be monitored after administration of a single intramuscular dose of MTX (50 mg/m^2^). A progressive decrease in serum human chorionic gonadotropin levels (at least 15%) is expected between days 4 and 7 post-injection [15]. If the serum β-hCG level falls by less than 15%, a second dose of MTX is required. Alternative protocols consist of either MTX multiple administration or local injection into the gestational sac MTX. In the literature, single mifepristone administration together with MTX has been shown to increase the success rate of medical therapy [16]. MTX is the most widely used chemotherapeutic agent, firstly described in 1982, it is the non-surgical treatment of choice for selected ectopic pregnancies [17,18]. MTX is a folic acid antagonist that prevents DNA synthesis in rapidly dividing cells at the implantation site. Mifepristone is a competitive progesterone receptor antagonist that causes trophoblast detachment from the uterine decidua, degeneration of the corpus luteum and induction of uterine contraction. MTX and mifepristone act synergistically and promote lysis of the trophoblast cells [19]. Alternatives to medical therapy are mentioned in the literature, namely, hysteroscopy combined with medical treatment [15,20], laparoscopic cornual resection [21], laparoscopic cornuostomy [22,23], and in the more severe cases laparotomic hysterectomy [24]. Minimally invasive surgery is indicated for elective procedures. Laparotomy may be indicated in patients with hemodynamic instability and hysterectomy may be required for otherwise uncontrollable hemorrhage [24,25]. Nevertheless, none of them have proven to be the best option. Considering the patient’s persistent haemodynamic condition and her strong desire to preserve her fertility, we opted for medical therapy, i.e., we administered a double dose of MTX in combination with mifepristone. However, when the clinical conditions changed and haemodynamic instability occurred, surgery was required to remove the ectopic pregnancy and eliminate the complications. However, the uterus was saved.

## 4. Conclusions

Interstitial pregnancy is a rare clinical condition. Medical therapy as a first approach is a feasible option; however, in the case of failure of surgical treatment, it should be the alternative [25,26]. An adequate and thorough medical and obstetric history is required to assess all the risks and benefits of either therapy or, after discussion with experts, to choose the most appropriate solution for the patient’s health. In our clinical case, we firstly chose medical therapy that showed great effectiveness, as no embryonal cardiac activity was detected and serum β-hCG levels decreased early on. Afterwards, uterine wall rupture occurred, probably due to patient’s risk factors (previous salpingectomy following an extrauterine pregnancy). It is very important to emphasise that in an emergency such as our case, a lifesaving laparotomic approach was preferred in view of the patient’s complete haemodynamic instability. The laparoscopic approach is clearly preferable when clinical conditions permit. It is, therefore, necessary to carry out appropriate counselling with the patient, who must also be made aware of the possibility of an emergency laparotomy. Considering the clinical data and our experience, we can assert that medical therapy with methotrexate and mifepristone is a valid therapeutic choice for extrauterine pregnancies, even when serum β-hCG levels are high. Further studies are needed to establish a standardized protocol, and maybe a clinical score that can be useful in predicting the patients in which medical therapy could be most successful. In fact, advances in diagnostic techniques allow increasingly precise and faster diagnoses to be performed for IP. They have also enabled increasingly precise and tailored therapy to be introduced. Our medical approach was driven by the patient’s strong desire to preserve her fertility and the possibility to constantly monitor her clinical condition in a protected hospital environment.

## Data Availability

All data are presented in the present manuscript.
